# Psychological Distress in Parents and School-Functioning of Adolescents: Results from the World Trade Center Registry

**DOI:** 10.1007/s11524-017-0143-4

**Published:** 2017-03-20

**Authors:** Lisa M. Gargano, Tenzin Dechen, James E. Cone, Steven D. Stellman, Robert M. Brackbill

**Affiliations:** 1 New York City Department of Health and Mental Hygiene, 42-09 28th Street, 7th Floor, Long Island City, NY 11101 USA; 2 Department of Epidemiology, Mailman School of Public Health, Columbia University, New York, NY 10032 USA

**Keywords:** Adolescents, World Trade Center, Unmet healthcare need, School-functioning

## Abstract

Poor school-functioning can be indicative of parent and adolescent mental health and adolescent behavior problems. This study examined 472 adolescents enrolled in the World Trade Center (WTC) Health Registry, with a two-step path analysis, using regression-based models, to unravel the relationships between parent and adolescent mental health, adolescent behavior problems, and adolescent unmet healthcare need (UHCN) on the outcome school-functioning. WTC exposure was associated with UHCN and parental mental health was a significant mediator. There was no evidence that family WTC exposure was associated with UHCN independent of its effect on parental mental health. For the second path, after accounting for the effects of adolescent mental health, behavioral problems, and UHCN, there remained a significant association between parental mental health and school-functioning. Interventions for poor school-functioning should have multiple components which address UHCN, mental health, and behavioral problems, as efforts to address any of these alone may not be sufficient.

## Introduction

The terrorist attack on the World Trade Center (WTC) in New York City on September 11, 2001 (9/11) has had long-term mental and physical health consequences[1–4]. Among adults, exposure to the events of 9/11 has been associated with mental health conditions including post-traumatic stress disorder (PTSD) and depression as well as physical health conditions including asthma and cardiovascular disease[1, 5–7]. Studies of adult survivors enrolled in the World Trade Center Health Registry (Registry) showed that 9/11 exposure was associated with unmet healthcare need (UHCN) [5, 8], but corresponding studies of unmet healthcare needs among 9/11-exposed children are limited. In previous studies of 9/11-exposed children enrolled in the Registry, approximately 5% of children were reported to have UHCN [9, 10]. In the same studies, parental 9/11-related psychopathology was associated with child behavior problems. More generally, parental psychopathology, including depression and anxiety, has been shown to be a predictor of UHCN in children [11, 12, suggesting a potential relationship between 9/11 exposure, parental mental health, and adolescent unmet healthcare needs. If emotional and behavioral problems of adolescents are left untreated, they may result in ongoing and lifelong psychological, social, and economic problems for the adolescent, families, and society [13].

Schools are a social and learning environment that affects not only the academic achievements of students but also their present and future health and well-being [14]. School-functioning refers to a student’s ability to perform important functional activities that support or enable participation in the academic aspects, such as grade and attendance, and nonacademic aspects, such as social relationships, of an educational program [15]. These functional activities are considered to be the nonacademic aspects of a school program and differ significantly from academic activities (e.g., classroom and homework assignments) [16]. Poor school-functioning can reflect many life experiences, including lack of access to appropriate health care. A 1997 study of 1285 children ages 9–17 in four US cities found that adolescents with grades of C or D were more likely to have UHCN that those adolescents with school grades of As [11]. UHCN has been shown to predict school absence [17], and studies suggest that school attendance affects school achievement [18].

While academic achievement is among the most thoroughly studied social consequences of mental health problems [19], less is known about how different adolescent and parent factors influence the broader attribute of school-functioning. Mental health disorders, including PTSD and depression, are known to impact school performance [20, 21]. Behavior problems, including internalizing and externalizing behavior problems in adolescents, are also known to affect academic achievement [19, 22].

There is limited literature on the combined influence of different factors, such as parental mental health problems, behavior and mental health problems, and UHCN on adolescent school-functioning. Understanding the mechanisms of poor functioning as an outcome of trauma experienced by parents and their children would greatly improve our capacity to understand children’s response to trauma in general. Furthermore, findings from this line of research are the key to target and serve populations who are in need, by informing the design of appropriate interventions which can be quickly implemented in a post-mass violence context. To address this gap, the current study utilized the World Trade Center Health Registry (Registry) to examine school-functioning as the primary outcome and its association with 9/11 exposure, parental mental health, adolescent’s behavior and mental health problems, and UHCN.

## Methods

### Data Collection and Study Population

The Registry’s study design and enrollment methods have been described previously [1, 23]. Briefly, the Registry longitudinally follows a cohort of 71,434 individuals, including rescue and recovery workers, lower Manhattan residents who lived south of Canal Street on 9/11, students and staff at schools South of Canal street on 9/11, building occupants, and passersby south of Chambers Street on 9/11 [1, 23]. Children were eligible for the Registry if they resided or were enrolled in school in Manhattan south of Canal Street or were present south of Chambers Street on the morning of 9/11. Children <18 years of age were recruited through their parents, and/or active outreach to community organizations and schools. Schools south of Canal Street in Manhattan, including child care centers, nursery schools, and public and private schools with grades kindergarten through 12th (K–12), were contacted by mail and telephone. In particular, the New York City Department of Education endorsed the Registry project to families of 12,600 public school children initially through mailed and subsequently backpack letters, and several private schools provided lists of student names.

The current study is based on data obtained at Waves 1 (2003–2004) and 3 (2011–2012). For enrollees under 18 years of age, Wave 1 surveys were completed by a parent proxy who provided information about the child’s health and WTC exposure. At Wave 3, parents of adolescents completed a survey that provided data on their relationship to the adolescent, household composition and income, the parent’s own emotional health, and the adolescent’s health. Adolescents completed a separate questionnaire at Wave 3 about their behavior, for which a separate return envelope was provided for confidentiality. After excluding enrollees without both a parent and linked adolescent survey, the final sample size was 472 (36%) adolescents who had complete Wave 1 and Wave 3 surveys and had a complete Wave 3 parent survey. The protocol used in this study was approved by the Institutional Review Boards of the NYC Department of Health and Mental Hygiene and the Centers for Disease Control and Prevention.

### Study Variables

#### School-Functioning

On their Wave 3 survey, adolescents completed the five items from the School Functioning Scale from the Pediatric Quality of Life Inventory Version™ 4.0 (PedsQL™ 4.0) [24]: difficulty paying attention in class, forgetting things, keeping up with school, missing school because of not feeling well, and missing school because of medical appointments and hospitalizations. Adolescent enrollees were asked to rate how much of a problem each item has been during the past month (never a problem, almost never a problem, sometimes a problem, often a problem, almost always a problem) [24]. Scores range from 0 to 100; higher scores indicate better school-functioning.

#### Unmet Health Care Needs in the Past 12 Months

Data on UHCN were collected from the parent Wave 3 survey, including parents’ perceived unmet physical health care needs for their adolescents, which was signified by a response of “Yes” for the question: “During the last 12 months, was there ever a time when your adolescent needed health care for physical health problems, but didn’t receive it?” Parents’ perceived unmet mental health care needs for the adolescents were signified by a response of “Yes” for the question: “During the last 12 months, was there ever a time when your adolescent needed mental health care or counseling, but didn’t receive it?” We created a 3-level variable indicating 0 = “no need,” 1 = “physical or mental health need,” and 2 = “both mental and physical health need.”

#### Parent Mental Health Problems

On the parent Wave 3 survey, parental non-specific psychological distress (NPD) symptoms were measured using the Kessler 6 (K6) scale, a validated tool which measures psychological symptoms in the past 30 days. The K6 consists of six items scored on a scale of 1 (not at all) to 5 (extremely), with scores ranging from 0 to 24. Severe NPD was defined as a score of ≥12 [25].

#### Family WTC Exposure

Family WTC exposure was taken from the Wave 3 parent survey and was defined as having a family member (mother, father, sibling, grandparent, or any other family member) who was injured or killed in the attacks, or was in the WTC disaster and escaped. This exposure definition was previously used by Hoven et al. in a survey of New York City school children 6 months post-attack [26] and in Registry studies on of child enrollees at Waves 1 [23] and 2. [9, 10] 

#### Strengths and Difficulties Questionnaire

On their Wave 3 survey, adolescents completed the Strengths and Difficulties Questionnaire (SDQ), a brief behavioral screening questionnaire that asks about 25 attributes (both positive and negative) that are divided among five scales of five items each, creating domain scores for conduct problems, hyperactivity, emotional symptoms, peer problems, and pro-social behavior [27–29]. A total difficulties score (0–40) was computed by adding scores from the four problem subscales (conduct, hyperactivity, emotional, and peer problem domains) [27–29]. The domains were scored as normal (0–15), borderline (16–19), and abnormal (20–40) based on normative US data [30]. The SDQ total difficulties score has been shown to be a psychometrically sound measure of child mental health problems [27, 30].

#### Adolescent Mental Health Outcomes

Probable PTSD, depression, and agoraphobia were found by Hoven et al. to be highly associated with WTC exposure [26]. They were assessed on the Wave 3 adolescent survey using the Diagnostic Interview Schedule for Children (DISC) Predictive Scales (DPS) which have been used in previous WTC publications on children [9, 10, 26]. The assessment includes seven questions that are designed to evaluate stress-related functioning during the past 4 weeks; a scoring algorithm was used to determine “impaired functioning.” The assessment also includes eight symptom questions to evaluate stress, which refer to WTC disaster-related experiences. Probable PTSD was assumed if adolescents scored in the impaired functioning range and answered “yes” to at least five of the eight symptom questions suggesting re-experiencing, avoidance, or sense of a foreshortened future. Probable depression was measured using a nine-item scale from the DPS. Criteria for probable depression included a positive response to seven or more items and impaired function. Probable agoraphobia was measured using a six-item scale, and criteria for probable agoraphobia were a positive response to three or more items and impaired functioning. Because the symptoms of PTSD, depression, and agoraphobia overlap, we created a variable (adolescent mental health) reflecting how many of these conditions (0–3) an adolescent was experiencing at the time of survey completion.

### Statistical Analyses

All analyses were performed in SAS 9.2. Descriptive analyses examined the bivariate correlations between the study variables. In order to disentangle the components that contribute to poor school-functioning, we approached the problem in the following steps. First, using a regression-based model, we examined the mediating role of parental mental health in the association between WTC family exposure and adolescent UHCN (Model 1). The next step examined the mediating roles of adolescent’s UHCN, behavior problems, and mental health in the relationship between parental K6 score and adolescent school-functioning (Model 2). Covariates included were household income (Model 1) and adolescent age (Model 2). This statistical strategy [31] allowed for estimation and significance testing of the total and specific indirect (mediation) effects through bootstrapping. Bootstrapping generates an empirical representation of the sampling distribution of the indirect effect, from which a confidence interval can be generated [31]. Analyses were conducted using PROCESS, a conditional modeling macro that uses an ordinary least squares-based path analytical framework to test for both direct and indirect effects [31]. A bias-corrected bootstrap confidence interval for the indirect effect based on 10,000 bootstraps was calculated as recommended [31].

## Results

### Sample Characteristics

The majority of adolescents at Wave 3 were 0–4 years of age on 9/11 and non-Hispanic white race/ethnicity (Table 1). The mean school-functioning score was 76.5 (SD ±17.5). Thirty-nine adolescents (8.7%) had at least one unmet healthcare need, 32 (7.0%) had borderline SDQ scores, and 23 (5.0%) had abnormal SDQ scores. Twenty-eight (5.9%) adolescents screened positive for one mental health condition, 8 (1.7%) had two, and 5 (1.1%) had three mental health issues. Over one-third of the sample had a family WTC exposure with 2.1% having a family member injured (*n* = 10), 33.5% (*n* = 158) having a family member in the disaster and escaped unharmed, and 1.3% (*n* = 6) having a family member killed in the disaster. The majority of parents (62.5%) reported a household income of greater than $75,000 in 2010 and higher than high school education (85.2%). Fifty-six (11.9%) parents screened positive for NPD (Table 1).

**Table 1:** Characteristics of WTCHR adolescents and their parents (*n* = 472)

	Total *N* (%)
Adolescent characteristics	
Age at 9/11 (age at Wave 3)	
0–4 years (10–14 years)	298 (63.1)
5–8 years (15–18 years)	174 (36.9)
Gender	
Male	235 (49.8)
Female	237 (50.2)
Race/ethnicity	
White	253 (53.6)
Others	219 (46.2)
School-functioning (Wave 3)	
Mean (standard deviation)	76.5 (±17.5)
Unmet healthcare needs (Wave 3)	
0	410 (91.3)
1	30 (6.7)
2	9 (2.0)
SDQ scores (Wave 3)	
Normal (0–15)	402 (88.0)
Borderline (16–19)	32 (7.0)
Abnormal (20–40)	23 (5.0)
Mental health problems (Wave 3)	
0	431 (91.3)
1	28 (5.9)
2	8 (1.7)
3	5 (1.1)
Family WTC exposure^a^	
Yes	162 (34.3)
No	310 (65.7)
Parent characteristics (Wave 3)	
Household income (2010)	
≤$75,000	166 (37.4)
>$75,000	278 (62.6)
Parents education	
≤High school	65 (14.8)
>High school	374 (85.2)
Parent NPD	
Yes (K6 ≥ 12)	56 (11.9)
No	413 (88.1)

^a^Having a family member who was injured or killed in the attacks, or was in the WTC disaster and escaped unharmed

Bivariate associations between the study variables are presented in Table 2. Adolescent UHCN was positively associated with parental K6 score (*r* = 0.26; *p* < 0.01). There was a strong correlation between parental NPD and adolescent SDQ (*r* = 0.28; *p* < 0.01) and adolescent mental health conditions (*r* = 0.23; *p* < 0.01). As parental K6 score increased, the adolescent’s SDQ score or number of mental health conditions increased. Adolescent mental health and SDQ score were also positively correlated (*r* = 0.50; *p* < 0.01). School-functioning was negatively associated with older adolescent age (*r* = −0.15; *p* < 0.01), UHCN (*r* = −0.21; *p* < 0.01), SDQ score (*r* = −0.51; *p* < 0.01), adolescent mental health (*r* = −0.48; *p* < 0.01), and parental K6 score (*r* = −0.29; *p* < 0.01). Parental education and household income, both measures of socio-economic status, were highly correlated (*r* = 0.51; *p* < 0.01).

**Table 2:** Bivariate correlations of adolescent and parent characteristics

Variable	2.	3.	4.	5.	6.	7.	8.	9.
1. UHCN	0.26^**^	0.16^**^	0.12^*^	−0.21^**^	0.09	0.01	−0.22^**^	−0.20^**^
2. Parent K6 score	–	0.28^**^	0.23^**^	−0.29^**^	0.12^*^	−0.03	−0.02	−0.15^*^
3. SDQ score		–	0.50^**^	−0.51^**^	0.08	0.05	−0.05	−0.15^*^
4. Adolescent mental health			–	−0.48^**^	0.09	0.08	−0.03	−0.09^*^
5. School-functioning^a^				–	−0.05	−0.15^**^	0.06	0.06
6. WTC family exposure					–	−0.06	0.17^**^	0.17^**^
7. Age						–	−0.002	−0.09
8. Parent education							–	0.51^**^
9. Household income								–

^a^Only variable in which larger numbers are better; for others (UHCN, K6, SDQ, adolescent mental health, and WTC family exposure), bigger numbers mean a poorer outcome

^*^ *p* < 0.05; ^**^ *p* < 0.01

### Model 1: Test for Mediation by Parental Mental Health of the Relationship Between 9/11 Exposure and Adolescent UHCN

**Fig. 1:**
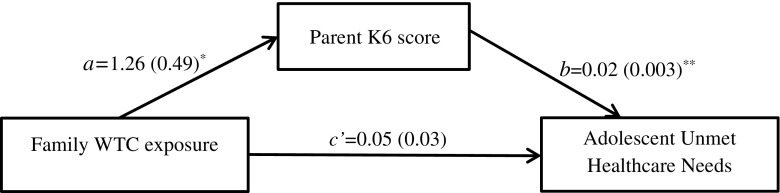
Mediation by parental mental health of the relationship between 9/11 exposure and adolescent UHCN controlling for household income. Standardized regression coefficients (standard errors); *p < 0.05, **p < 0.01

Figure 1 shows the results of the ordinary least squares path analysis in which parental NPD was hypothesized to be a mediator of a potential causal relationship between family WTC exposure and adolescent unmet healthcare needs. Family WTC exposure was associated with an increase in parental K6 score (*a* = 1.26, SE = 0.49, *p* = 0.01). Parental K6 score was positively associated with UHCN in adolescents (*b* = 0.02, SE = 0.003, *p* < 0.0001). A bias-corrected bootstrap confidence interval for the indirect effect (*ab* = 0.02, SE = 0.01) was statistically significant (0.005–0.05). There was no evidence that family WTC exposure was associated with adolescent UHCN independent of its effect on parental NPD (*c’* = 0.05, SE = 0.03, *p* = 0.17).

### Model 2: Test for Mediation by Adolescent’s Unmet Healthcare Needs, Adolescent Behavior Problems, and Adolescent Mental Health of the Relationship Between Parental Mental Health and Adolescent’s School-Functioning

**Fig. 2:**
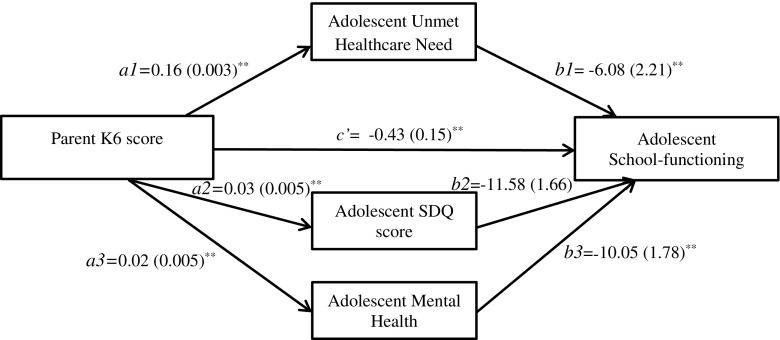
Mediation by adolescent’s unmet healthcare needs, adolescent behavior problems, and adolescent mental health of the relationship between parental mental health and adolescent’s school-functioning controlling for adolescent age. Standardized regression coefficients (standard errors); *p < 0.05, **p < 0.01

Figure 2 shows the results of the ordinary least squares path analysis in which adolescent UHCN, SDQ score, and mental health are hypothesized to be mediators of a potential causal relationship between parental K6 score and adolescent school-functioning. Parental K6 score was positively associated with adolescent UHCN (*a1* = 0.16, SE = 0.003, *p* < 0.0001), adolescent SDQ score (*a2* = 0.03, SE = 0.005, *p* < 0.0001), and adolescent mental health (*a3* = 0.02, SE = 0.005, *p* < 0.0001). Adolescent UHCN (*b1* = −6.08, SE = 2.21, *p* = 0.006), SDQ score (*b2* = −11.58, SE = 1.66, *p* < 0.0001), and mental health (*b3* = −10.05, SE = 1.78, *p* < 0.0001) were all associated with a decrease in school-functioning. A bias-corrected bootstrap confidence interval for the indirect effect for each variable (*ab1* = 10.10, SE = 0.05; *ab2* = −0.32, SE = 0.10; *ab3* = −0.24, SE = 0.08) was entirely below zero (UHCN (−0.22, −0.02); SDQ (−0.53, −0.17); mental health (−0.43, −0.11)). In this model, there is evidence that the effect of parental K6 on adolescent school-functioning is mediated by the adolescent’s UHCN, SDQ score, and mental health. In addition, parental K6 score remained independently associated with school-functioning even when taking the other variables into account (*c’* = −0.43, SE = 0.15, *p* = 0.005).

## Discussion

The WTC disaster had a cascading effect on families directly affected by the disaster. In this study, we used school-functioning as a measure of adolescent well-being and showed that “family exposure” to the disaster influenced adolescent school-functioning up to 10 years both directly and indirectly through a combination of parental mental health and adolescent behavior and mental health. In the present study, the mean school-functioning score of 9/11-exposed adolescents was similar to published reports on the general population (mean score 77.3) [24, 32].

School is centrally important to the lives of adolescents, with school attendance and performance representing the “work” of this developmental stage. Many internal and external factors affect the development of children’s school-functioning. These factors include cognitive traits (i.e., language development, pre-literacy skills) but more important for this study, non-cognitive traits, including self-control strategies and social skills that facilitate children’s success in a structured school learning environment [33]. Studies consistently find that children who are “unready” behaviorally are at much greater risk of academic failure than other children [34]. Another study found that children who were better engaged in school were more likely to go on to a professional, semi-professional, or managerial career [35].

Our study demonstrated that there are two stages of influence on school-functioning after a disaster. The first stage is reflected by the effect of disaster trauma on parental mental health and also simultaneously on adolescent mental health and behavior. Although PTSD, depression, and other mental health consequences have been extensively documented in adults following the 9/11 terrorist attacks [5, 6], there is a limited literature that has focused on the estimated 25,000 children who were also directly exposed to the disaster. We are unaware of any prior studies that have examined the long-term effect of 9/11 on adolescent behavior and mental health. Nonetheless, studies that were conducted within 18-month post-9/11 suggest a wide range of serious 9/11-related mental health and behavioral outcomes affecting children and adolescents [9, 26, 36, 37]. One study conducted 6 months after 9/11 found 29% of NYC school children had one or more of six probable mental health disorders, with almost 11% having probable PTSD [26]. Another study of high school students in Bronx found that 8 months after 9/11, 7% had PTSD symptom cluster [36], while 18 months after 9/11, the prevalence of PTSD in middle and high schools near Ground Zero was 4% and depression was just under 5% [37]. Normative prevalence data for PTSD among adolescents in New York City prior to 9/11 are limited, but performance testing of the DISC Version 2.3 in the early 1990s in four cities including New York City found a prevalence of 1.6% for any anxiety disorder using DSM-III-R criteria with diagnosis-specific impairment criteria [38].

The second stage of influence on school-functioning after a disaster represents the impact of parental mental health on the adolescent’s mental health and behavior. Most notably, adolescent 9/11-related PTSD has been shown to be significantly associated with 9/11-related PTSD in the parent [9]. Other relationships between child behavior and parental stress due to 9/11 have been reported in a number of studies. For example, maternal depression and PTSD were associated with increased emotional reactivity and aggressive behavior problems in their preschool children [39]. Among adolescents, parental 9/11-related PTSD was also associated with total SDQ scores and internalizing subscales [9]. Another study found that WTC workers with probable PTSD were more likely to report psychological symptoms and behavioral problems in their children compared with WTC workers without probable PTSD [40].

Other research outside the disaster setting has documented the importance of parental help-seeking behavior in receipt of mental health services for children who need them [41, 42]. A growing body of evidence suggests that parental mental health may impact their decisions about their child’s health care [43, 44]. One study found high levels of PTSD among injured children and their parents after automobile accidents, and that the parents’ symptoms affected decisions to provide appropriate care for their children [45]. Parents thus play a critical role in facilitating access to professional services for adolescents with emotional or behavioral problems.

Finally, parental mental health, adolescent mental health and behavior, and adolescent UHCN were all found to impact the adolescent’s school-functioning. Consistent with prior findings, parental psychopathology is associated with adolescent school-functioning [11, 12]. Beyond the parental factor, we found that adolescent factors, specifically, probable mental health conditions, behavioral issues, and UHCN, are part of the pathway in the relationship between parental mental health status and school-functioning, but they do not explain the entire relationship. There remains a direct effect of parental mental health on adolescent school-functioning, and/or other factors that were not measured account for the direct effect.

These findings are consistent with published reports that each of these factors have been shown to impact school-functioning [11, 19–22] but few studies consider multiple problems simultaneously [46]. Many adolescents experience more than one problem, [47]; thus, studies that focus on single problem are limiting and may produce biased estimates, attributing associations to only one condition. The strength of this study is that it incorporated more than one factor in the system and estimated relative contribution by the different factors on school-functioning. Addressing any one of the factors alone in an intervention or policy decision that includes UHCN, mental health, behavioral problems, and poor school-functioning may not be sufficient. In addition, with training, teachers may be able to act to potentially identify students in need of care, particularly after a disaster.

In 2009, the Registry, with community and academic input, published clinical guidelines for pediatricians and other medical personnel treating children and adolescents who may have been affected by 9/11 experiences [48]. Interventions to improve school-functioning among adolescents should include mental health and behavioral components for the adolescent as well as components to address potential family factors. Our findings suggest that school teachers and counselors should be aware that impairments in school-functioning, such as poor grades and attendance or inattentiveness, could be reflections of the young persons’ own mental health, other behavior problems, or their unmet health care needs that could be related to parental mental health issues.

### Limitations

This current study has several limitations including its reliance on self-reported WTC exposures and parental NPD, which may be subject to response bias. To assess unmet health care needs, we asked parents about their perceptions of an adolescent’s unmet need for care. This approach has been used in several studies of children’s access to care [49, 50]. Although commonly used, this approach is subject to recognition and recall error. Additionally, we found that parents who did not complete the Wave 3 survey differed significantly on race and income in 2002 compared to those who completed Wave 3 which may have biased our results. The relatively low response rate (∼36%) and resulting small sample size may have resulted in reduced power for hypotheses affecting certain subgroups.

## Conclusions

Post-disaster education is needed for healthcare providers of both adolescents and parents on the relationship between parental mental health and the healthcare decisions that parents make on behalf of their adolescent. It is important to find effective ways to reduce barriers and facilitate access for appropriate help and support for young people with early signs and symptoms of common mental disorders as well as behavior problems.

## References

[1] Brackbill RM, Hadler JL, DiGrande L (2009). Asthma and posttraumatic stress symptoms 5 to 6 years following exposure to the World Trade Center terrorist attack. JAMA.

[2] Li J, Cone JE, Alt AK (2016). Performance of self-report to establish cancer diagnoses in disaster responders and survivors, World Trade Center Health Registry, New York, 2001–2007. Public Health Rep.

[3] Perlman SE, Friedman S, Galea S (2011). Short-term and medium-term health effects of 9/11. Lancet.

[4] Welch AE, Caramanica K, Maslow CB, Brackbill RM, Stellman SD, Farfel MR (2016). Trajectories of PTSD among Lower Manhattan residents and area workers following the 2001 World Trade Center disaster, 2003–2012. J Trauma Stress.

[5] Brackbill RM, Stellman SD, Perlman SE, Walker DJ, Farfel MR (2013). Mental health of those directly exposed to the World Trade Center disaster: unmet mental health care need, mental health treatment service use, and quality of life. Soc Sci Med.

[6] Caramanica K, Brackbill RM, Liao T, Stellman SD (2014). Comorbidity of 9/11-related PTSD and depression in the World Trade Center Health Registry 10–11 years postdisaster. J Trauma Stress.

[7] Jordan HT, Stellman SD, Morabia A (2013). Cardiovascular disease hospitalizations in relation to exposure to the September 11, 2001 World Trade Center disaster and posttraumatic stress disorder. J Am Heart Assoc.

[8] Ghuman SJ, Brackbill RM, Stellman SD, Farfel MR, Cone JE (2014). Unmet mental health care need 10–11 years after the 9/11 terrorist attacks: 2011–2012 results from the World Trade Center Health Registry. BMC Public Health.

[9] Mann M, JLi J, Maslow CB, Osahan S, Stellman SD (2015). Adolescent behavior and PTSD 6–7 years after the World Trade Center terrorist attacks of September 11, 2001. Disaster Health.

[10] Stellman SD, Thomas PA, Osahan S, Brackbill RM, Farfel MR (2013). Respiratory health of 985 children exposed to the World Trade Center disaster: report on world trade center health registry wave 2 follow-up, 2007–2008. J Asthma.

[11] Flisher AJ, Kramer RA, Grosser RC (1997). Correlates of unmet need for mental health services by children and adolescents. Psychol Med.

[12] Jensen PS, Bloedau L, Davis H (1990). Children at risk: II. Risk factors and clinic utilization. J Am Acad Child Adolesc Psychiatry.

[13] Ryan SM, Jorm AF, Toumbourou JW, Lubman DI (2015). Parent and family factors associated with service use by young people with mental health problems: a systematic review. Early Interv Psychiatry.

[14] Anderman LH, Freeman TM. “Students' sense of belonging in school”. In: Maehr M, Pintrich P, editors. Advances in motivation and achievement: motivating students, improving schools: the legacy of Carol Midgley, Vol. 13. Greenwich, CT: JAI Press; 2004. p 27–63.

[15] Dick BD, Pillai Riddell R (2010). Cognitive and school functioning in children and adolescents with chronic pain: a critical review. Pain Res Manag.

[16] Coster WJ, Deeney TA, Haltiwanger JT, Haley SM (2008). School Function Assessment: Technical Report.

[17] Stipp KF (2012). Access to Health Care and School Engagement in U.S. School Children.

[18] Case A, Paxson C (2006). Children’s health and social mobility. Future Child.

[19] McLeod JD, Uemura R, Rohrman S (2012). Adolescent mental health, behavior problems, and academic achievement. J Health Soc Behav.

[20] Bhatia SK, Bhatia SC (2007). Childhood and adolescent depression. Am Fam Physician.

[21] Schwarz E, Perry BD (1994). The post-traumatic response in children and adolescents. Psychiatr Clin N Am.

[22] McLeod JD, Kaiser K (2004). The relevance of childhood emotional and behavioral problems for subsequent educational attainment. Am Sociol Rev.

[23] Thomas PA, Brackbill R, Thalji L (2008). Respiratory and other health effects reported in children exposed to the World Trade Center disaster of 11 September 2001. Environ Health Perspect.

[24] Varni JW, Burwinkle TM, Seid M (2006). The PedsQL 4.0 as a school population health measure: feasibility, reliability, and validity. Qual Life Res.

[25] Kessler RC, Andrews G, Colpe LJ (2002). Short screening scales to monitor population prevalences and trends in non-specific psychological distress. Psychol Med.

[26] Hoven CW, Duarte CS, Lucas CP (2005). Psychopathology among New York City public school children 6 months after September 11. Arch Gen Psychiatry.

[27] Bourdon KH, Goodman R, Rae DS, Simpson G, Koretz DS (2005). The Strengths and Difficulties Questionnaire: U.S. normative data and psychometric properties. J Am Acad Child Adolesc Psychiatry.

[28] Goodman R (2001). Psychometric properties of the strengths and difficulties questionnaire. J Am Acad Child Adolesc Psychiatry.

[29] Richter J, Sagatun A, Heyerdahl S, Oppedal B, Roysamb E (2011). The Strengths and Difficulties Questionnaire (SDQ)—self-report. An analysis of its structure in a multiethnic urban adolescent sample. J Child Psychol Psychiatry.

[30] Anmyr L, Larsson K, Olsson M, Freijd A (2012). Strengths and difficulties in children with cochlear implants—comparing self-reports with reports from parents and teachers. Int J Pediatr Otorhinolaryngol.

[31] Hayes AF (2013). Introduction to Mediation, Moderation, and Conditional Process Analysis.

[32] Varni JW, Burwinkle TM, Seid M, Skarr D (2003). The PedsQL 4.0 as a pediatric population health measure: feasibility, reliability, and validity. Ambul Pediatr.

[33] Farkas G (2003). Cognitive skills and noncognitive traits and behaviors in stratification processes. Annu Rev Sociol.

[34] La Paro KM, Pianta RC (2000). Predicting children’s competence in the early school years: a meta-analytic review. Rev Educ Res.

[35] Abbott-Chapman J, Martin K, Ollington N, Venn A, Dwyer T, Gall S (2013). The longitudinal association of childhood school engagement with adult educational and occupational achievement: findings from an Australian national study. BERJ.

[36] Calderoni ME, Alderman EM, Silver EJ, Bauman LJ (2006). The mental health impact of 9/11 on inner-city high school students 20 miles north of Ground Zero. J Adolesc Health.

[37] Chemtob CM, Nomura Y, Josephson L, Adams RE, Sederer L (2009). Substance use and functional impairment among adolescents directly exposed to the 2001 World Trade Center attacks. Disasters.

[38] Shaffer D, Fisher P, Dulcan MK (1996). The NIMH Diagnostic Interview Schedule for Children Version 2.3 (DISC-2.3): description, acceptability, prevalence rates, and performance in the MECA Study. Methods for the Epidemiology of Child and Adolescent Mental Disorders Study. J Am Acad Child Adolesc Psychiatry.

[39] Chemtob CM, Nomura Y, Rajendran K, Yehuda R, Schwartz D, Abramovitz R (2010). Impact of maternal posttraumatic stress disorder and depression following exposure to the September 11 attacks on preschool children’s behavior. Child Dev.

[40] Stellman JM, Smith RP, Katz CL (2008). Enduring mental health morbidity and social function impairment in world trade center rescue, recovery, and cleanup workers: the psychological dimension of an environmental health disaster. Environ Health Perspect.

[41] Feehan M, Stanton W, McGee R, Silva PA (1990). Parental help-seeking for behavioural and emotional problems in childhood and adolescence. Commun Health Stud.

[42] Pavuluri MN, Luk SL, McGee R (1996). Help-seeking for behavior problems by parents of preschool children: a community study. J Am Acad Child Adolesc Psychiatry.

[43] Bethell C, Peck C, Schor E (2001). Assessing health system provision of well-child care: the Promoting Healthy Development Survey. Pediatrics.

[44] Janicke DM, Finney JW, Riley AW (2001). Children’s health care use: a prospective investigation of factors related to care-seeking. Med Care.

[45] de Vries AP, Kassam-Adams N, Cnaan A, Sherman-Slate E, Gallagher PR, Winston FK (1999). Looking beyond the physical injury: posttraumatic stress disorder in children and parents after pediatric traffic injury. Pediatrics.

[46] Breslau J, Lane M, Sampson N, Kessler RC (2008). Mental disorders and subsequent educational attainment in a US national sample. J Psychiatr Res.

[47] Costello EJ, Mustillo S, Erkanli A, Keeler G, Angold A (2003). Prevalence and development of psychiatric disorders in childhood and adolescence. Arch Gen Psychiatry.

[48] Cone JE, Perlman SE, Eros-Sarnyai M (2009). Clinical guidelines for children and adolescents exposed to the World Trade Center disaster. City Health Inf.

[49] Lave JR, Keane CR, Lin CJ, Ricci EM, Amersbach G, LaVallee CP (1998). Impact of a children’s health insurance program on newly enrolled children. JAMA.

[50] Simpson G, Bloom B, Cohen RA, Parsons PE (1997). Access to Health Care. Part 1: Children. Series 10. Data from the National Health Survey.

